# Research on the stability of the ballast water tank on the offshore floating platform based on numerical simulation

**DOI:** 10.1371/journal.pone.0334494

**Published:** 2025-10-16

**Authors:** Huiyuan Zheng, Shicheng Wang, Shihua Li, Xin Wang, Kuan Lu, Dianhu Li, Lan Xia, Yunzhou Li, Bilin Wu

**Affiliations:** 1 National Ocean Technology Center, Tianjin, China; 2 The Key Laboratory of Ocean Observation Technology of the Ministry of Natural Resources, Tianjin, China; 3 Institute of Oceanographic Instrumentation, Qilu University of Technology (Shandong Academy of Sciences), Qingdao, Shandong, China; 4 School of Marine Science and Technology, Northwestern Polytechnical University, Xi’an, Shaanxi, China; Tongji University, CHINA

## Abstract

The study of stabilisation performance is a crucial consideration in the design of offshore floating platforms. For large floating structures, incorporating passive anti-roll tanks is a common technique for roll reduction. To investigate the feasibility of using ballast tanks for roll attenuation on the “Guo Hai Shi 1” platform, this study employs the internal tank theory to analyze the influence of four independent empty tanks, located around the platform, acting as anti-roll tanks with varying ballast water volumes. The results indicate that: (1) Different ballast water volumes within a single tank do not significantly affect the static stability parameters of the platform. (2) In regular wave simulations, the ballast tanks show limited effectiveness in reducing pitch motion along the wave incidence direction but effectively suppress coupled responses in other degrees of freedom. In the resonance case (Case 3), the minimum pitch occurs in Condition 1 at 10.56°, while the maximum pitch reaches 11.45° in Condition 2. Nevertheless, a 40% reduction in roll motion is achieved (3.36° in Condition 4 vs. 5.60° in Condition 1), along with a 24.5% reduction in yaw motion (39.22° in Condition 4 vs. 51.94° in Condition 1). (3) In irregular wave simulations, the ballast tanks effectively reduce the heave amplitude by up to 8.34% in sea state level 4 and 6.06% in sea state level 8, thereby enhancing its wave-following performance in the heave degree of freedom. (4) A CNN_BiLSTM_Attention algorithm is developed using hydrodynamic analysis generated datasets to predict the pitch motion time series of the platform under different ballast water conditions and sea states, while the model has a superior prediction performance (R² = 0.9658, RMSE = 0.5343, MAE = 0.3188, representing a 4.82% increase in R² and 30.31% reduction in RMSE compared to the original model). Future work will further explore the application of ballast tanks on floating platforms, with a focus on performance optimization and the development of advanced neural network models capable of predicting motion responses under various ballast configurations. Moreover, appropriate evaluation metrics will be established to assess the effectiveness of ballast tank designs. Efforts will also be directed towards integrating time-domain motion prediction using neural networks with control theories aimed at dynamically regulating ballast water volume to enhance platform stability.

## Introduction

In modern conventional medium and large-scale offshore floating platforms, ballast water management systems are widely implemented to monitor and control the loading, discharge, and treatment of ballast water. These systems enable precise water volume regulation and distribution through automated control mechanisms, ensuring optimal anti-roll or stabilisation performance under various sea conditions, which also serve as anti-roll tanks [1]. Based on different approaches to ballast water management for roll reduction, ballast tanks can be generally classified into three categories: active, passive, and controllable passive anti-rolling tanks [2].

Typically, ballast water tanks are distributed within the double-bottom compartments, forepeak tanks, aft peak tanks, or deepwater tanks [3]. Similarly, the “Guohai Shi 1” floating offshore platform, part of the National Marine Test Site (Weihai), is equipped with four forepeak and aft peak tanks distributed across the bow and stern sections of its two demi-bodies, serving as passive anti-rolling tanks.

“Guo Hai Shi 1” is a significant multifunctional marine comprehensive experimental platform in China, specifically designed for marine scientific research and technological development validation. The platform is equipped with advanced marine testing instruments and comprehensive living facilities, enabling a wide range of offshore experimental applications, including marine environmental monitoring, ocean energy development, and marine equipment testing. It aims to provide experimenters with a stable, safe, and efficient research environment. Consequently, the study of the impact of ballast water tanks on the platform’s stability has been a crucial research focus throughout its construction and subsequent maintenance, which also provides design reference ideas for the subsequent design of new test platforms.

Anti-roll tanks play a critical role in ensuring the stability and safe operation of floating offshore platforms under various marine environmental conditions. By actively regulating water intake and discharge, these tanks adjust the buoyancy and draft of the platform, thereby maintaining its stability across different loading conditions [4]. Offshore platforms are typically subjected to significant environmental forces, including wind, waves, and tidal currents [5]. Proper water management of the tank enhances platform inertia and stabilisation moments, thereby reducing tilting and oscillations. This improves the platform’s resistance to wind and wave impacts, effectively mitigating response amplitude and frequency under extreme sea states to ensure safe and stable operations [6]. Recent advancements highlight growing integration of active control systems and multiphysics modeling for ballast optimization. For instance, Liu et al. (2025) established a novel fluid-structure coupling framework for floating wind turbines, demonstrating how ballast dynamics significantly influence platform motions – a critical insight for stability-sensitive operations [7]. Similarly, Zhang et al. (2025) validated active anti-rolling fluid momentum wheels in cylindrical FPSOs, showcasing emerging trends in adaptive stabilization technologies under wave excitation [8]. Furthermore, the role of passive anti-rolling tanks has also been analysed in a number of research papers, for example:

Liang Lihua et al. investigate the design principles and applications of a variable period passive stabilizing water tank for offshore platforms. A mathematical model of the tank is developed, and simulations are conducted to analyze its stabilizing performance under different operational conditions. The results demonstrate that the water tank significantly improves platform stability and shows good adaptability in varying sea states, providing a theoretical foundation and engineering reference for the design of offshore platform anti-wind and wave systems [9].

Alujević et al. investigated the impact of U-shaped anti-rolling tanks on ship rolling motion, analyzing the absorption and dissipation of wave-induced excitation through the interaction between the tank and rolling motion and reducing roll amplitude under various wave conditions, offering insights into its design and performance [10].

Bernal-Colio et al. conducted tank experiments using a swing table, recording the effects of different internal structures on tank performance. Based on these experimental results, they carried out numerical simulations to evaluate the accuracy of several computational models and the effectiveness in stabilizing vessel motions, providing valuable insights for anti-roll tank design [11].

However, current research predominantly focuses on conventional tank geometries, with limited studies addressing irregular configurations or real-time stability control—a gap critical for platforms like “Guo Hai Shi 1” where structural complexity compromises traditional stability analysis models. Unlike other passive anti-rolling tanks, the ballast water tanks of “Guo Hai Shi 1” are not the commonly used U-shaped or rectangular anti-rolling tanks typically positioned at the platform’s central area. Instead, they are composed of a combination of semi-circular, rectangular, and irregular wedge-shaped sections. The four ballast tanks are not interconnected, and the roll reduction effect of an individual tank’s water level is difficult to represent linearly.

To investigate the anti-rolling effect, this study adopts a quantitative approach by adjusting the ballast water volume. A hydrodynamic modeling and simulation analysis is conducted to examine the sensitive frequencies of the platform’s wave response. By varying the water volume in proportion to the platform’s displacement, the study evaluates the stabilisation performance under different environmental conditions and ballast water ratios. Through an assessment of the platform’s operational environment and motion response characteristics, the results provide insights into the effectiveness of ballast water tanks in roll reduction. This addresses a key engineering problem: optimizing non-standard tank configurations for stability in small-to-medium platforms, where traditional design heuristics may fail in such scenarios, and offering a reference for the design of ballast water tanks for our new floating platforms while laying the foundation for subsequent research on their impact on platform stability.

Finally, a CNN_BiLSTM_Attention deep learning model is deployed to predict pitch motion time series, serving as a bridge between simulation insights and operational safety. This enables proactive stability management by forecasting motion exceedances beyond safety thresholds, thereby supporting real-time ballast adjustments. The approach establishes a framework linking numerical stability analysis with control system—a critical step toward resilient platform operations. However, this paper only demonstrates the feasibility of the algorithm, the specific research content of which will be discussed in detail in future articles. From this, by coupling detailed hydrodynamic modeling with data-driven forecasting and control, this work provides a comprehensive methodology for stabilisation management on floating offshore platforms, guiding future platform design and real-time operations.

## Methodology

### Internal tank method

Under ideal conditions, an anti-rolling tank achieves roll reduction by generating a periodic restoring moment through the oscillatory motion of the internal fluid, counteracting the external moments acting on the platform [12]. The fundamental design principle of anti-rolling tanks is based on the concept of “dual resonance”, where the natural period of the platform matches the oscillation period of the fluid inside the tank [13]. When the platform undergoes roll or pitch motion due to wave-induced moments, resonance occurs if the platform’s natural period is close to the wave period, leading to significant motion amplitudes. At this stage, the platform’s oscillation phase lags the wave motion by 90°. Simultaneously, the fluid inside the anti-rolling tank also resonates, but due to fluid inertia, its motion lags behind the platform’s movement by another 90°. As a result, the motion of the internal fluid is 180° out of phase with the wave-induced excitation. This phase difference causes the restoring moment generated by the variation in the internal fluid level to counteract the wave-induced moment, thereby reducing the platform’s motion response amplitude [14].

However, if the restoring moment generated by the anti-rolling tank has no phase difference with the excitation moment induced by wave loading, or if the phase lag is only 90°, the roll reduction effect becomes insignificant. Moreover, if the phase lag of the restoring moment exceeds 180° relative to the platform’s motion, the tank may not only fail to provide roll reduction but could even exacerbate rolling, leading to an amplified motion response. Therefore, the proper design and utilization of ballast water tanks are critical to ensuring their effectiveness in anti-rolling stabilization.

**Fig 1:**
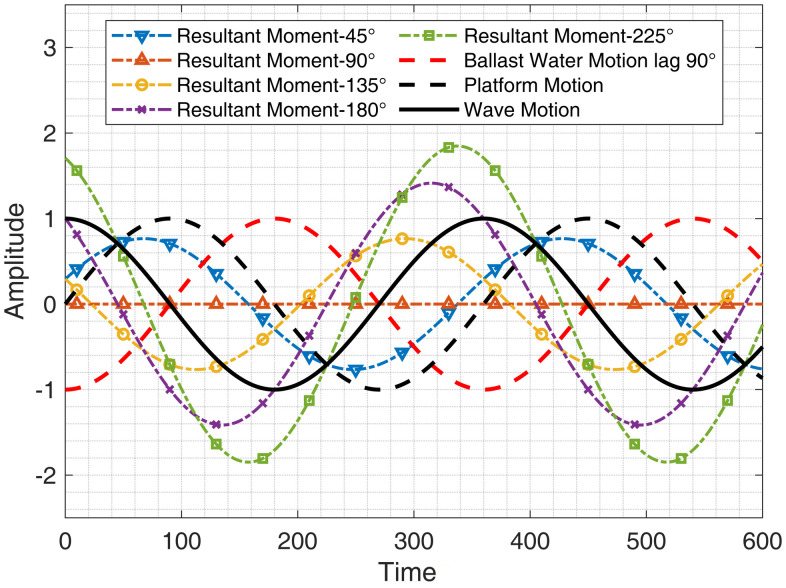
Resultant moment diagram.

The following figure shows the phase relationship between the wave motion, the platform response motion and the water movement of the ballast tank, and the water movement is ideally lagging behind the platform motion by 1/4 cycle. Assuming that the wave excitation moment and the restoring moment generated by the ballast water tank are equal in amplitude, then the two cancels out and the actual ideal stable resultant moment is 0. However, if it is not in the ideal state, then the actual resultant moment synthesised by the two is not 0, and according to the difference of the phase lag, it will play a different effect of slightly decreasing the combined moment or increasing the combined moment as shown in the Fig 1.

For the “Guo Hai Shi 1” platform, the design of its internal tanks is already fixed and difficult to modify. Therefore, if these tanks are to be utilized as anti-rolling devices, a more detailed investigation into the interactions between the tanks, the platform, and the wave environment is necessary. Analyzing whether the existing ballast tanks can achieve effective roll reduction will provide valuable insights for future platform construction, offering design references for optimizing ballast tank configurations.

The hydrodynamic analysis is used to simulate the interaction between the ballast water tanks and the platform’s motion by modeling the internal tank dynamics. The sloshing motion of the fluid inside the tank is strongly coupled with the motion of the platform in waves. Under the assumption of linear potential flow theory—similar to the wave resistance analysis of floating structures—the sloshing motion of the internal fluid is modeled with the assumptions that the fluid is inviscid, incompressible, and irrotational, and that the motion amplitude remains small.

In this model, the internal fluid does not exhibit incident or diffracted wave potential components. In the frequency domain, within the LTA coordinate system (Local Set of Axes), the total potential of the internal fluid consists of the sloshing motion components induced by the six-degree-of-freedom motion of the platform.


$$\begin{array}{c} \Phi \left({\vec{x}}_{t},t\right)=\phi \left({\vec{x}}_{t},\omega \right){\text{e}}^{-\text{i}\omega t}=\sum_{\text{j}=\text{1}}^{\text{6}}x_{tj}\phi_{j}\left({\vec{x}}_{t},\omega \right){\text{e}}^{-\text{i}\omega t} \end{array}$$ (1)

where *ω* is the frequency of the harmonic motion of the floating body, $\phi \left({\vec{x}}_{t},\omega \right)$ is the isolated spatial dependence term due to the jth fundamental motion of the water body in the internal tank of unit amplitude, $x_{tj}$ is the response amplitude operator (RAO) for the jth fundamental motion in the tank.

The potential energy of the water body in the internal tank is given by the following equation, in the fluid domain, ${\vec{x}}_{t}=\Omega_{t}$


$$\Delta \phi_{j}=\left(\frac{\partial^{\text{2}}}{\partial {\text{x}}^{\text{2}}}+\frac{\partial^{\text{2}}}{\partial {\text{y}}^{\text{2}}}+\frac{\partial^{\text{2}}}{\partial {\text{z}}^{\text{2}}}\right)\phi_{j}=\text{0}$$ (2)

On the average liquid-free surface of the LTA, $z_{t}=\text{0}$


$$-\nu \phi_{j}+\frac{\partial \phi_{j}}{\partial \text{z}}=\left\{ \;\begin{array}{cc} \text{0}, & \left(j\neq \text{3}\right)\\ -\text{i}\omega , & \left(j=\text{3}\right) \end{array}\right.$$ (3)

Where $\text{v}=\omega^{\text{2}}/g$, the average free surface of the water in the formula moves along the vertical rigidity with the vertical lifting motion of the platform and the water cabin, and this degree of freedom motion does not cause the vertical displacement of the centre of buoyancy of the water body in the internal tank.

The potential energy of the internal fluid is computed using the boundary integral method based on the source representation method. In this method, fluid motion is simulated by distributing source points on the surface of the floating body and its vicinity. Each source point generates a radiated wave field, and the overall fluid flow around the structure is represented as the superposition of these wave fields. By solving the Laplace equation, the source intensity distribution distribution is determined to satisfy the boundary conditions of the free surface, ensuring that the velocity at the floating body’s surface matches its motion velocity.

The deep-water pulsating source Green’s function is employed, and the velocity potential is expressed as follows:


$$\phi_{j}\left({\vec{x}}_{t},\omega \right)=\frac{\text{1}}{4\pi }\int_{{\text{S}}_{t}}\sigma_{j}\left({\vec{\xi }}_{t},\omega \right)\text{G}\left({\vec{x}}_{t},{\vec{\xi }}_{t},\omega \right)\text{dS}$$ (4)

Where ${\vec{x}}_{t}\in S_{t}\cup \Omega_{t},{\vec{\xi }}_{t}\in S_{t},j\neq \text{3}$, ${\vec{\xi }}_{t}$ is a certain source point.

Since the fluid is considered inviscid in potential flow theory, the resonance intensity of the internal fluid may be overestimated during the calculation process. Therefore, a damping correction is required for the boundary conditions. The source strength on the wetted surface of the tank is determined by the following modified boundary conditions, where the original boundary condition is given as:


$$\frac{\partial \phi_{j}}{\partial \text{n}}=-\text{i}\omega n_{j}$$ (5)

The modified boundary condition is given by


$$\frac{\partial \phi_{j}}{\partial \text{n}}+\text{i}\alpha_{t}{\text{f}}_{t}\left(\nu ,{\text{k}}_{\text{0}}\right)\phi_{j}=-\text{i}\omega n_{j},(j\neq \text{3})$$ (6)

$${\text{f}}_{t}\left(\nu ,{\text{k}}_{\text{0}}\right)=\left\{ \;\;\begin{array}{c} {\text{k}}_{\text{0}}{\text{sin}}^{\text{2}}\left(\frac{\pi }{\text{2}}\frac{\nu }{{\text{k}}_{\text{0}}}\right)\text{where}\nu <{\text{k}}_{\text{0}}\\ {\text{k}}_{\text{0}}\text{where}\nu <{\text{k}}_{\text{0}} \end{array}\right.$$ (7)

$${\text{k}}_{\text{0}}=\frac{\pi }{\text{max}(\text{L},\text{B})}\text{tanh}\left[\frac{\pi \text{T}}{\text{max}(\text{L},\text{B})}\right]$$ (8)

Here, $\alpha_{t}$ represents a positive dimensionless damping coefficient, while $k_{\text{0}}$ denotes the empirical approximation of the minimum sloshing resonance wavenumber inside the tank. For a rectangular tank with dimensions (L,B,T)—where L and B are the length and width of the tank, and T is the draft of the internal fluid. In practical computations, a viscous damping factor is typically introduced, with its value generally ranging between 0 and 0.1. The precise value can be calibrated through CFD simulations or experimental measurements. In this study, the damping factor is set to 0.05.

The source intensity equation is given by:


$$-\frac{\text{1}}{\text{2}}\sigma_{\text{j}}\left({\vec{x}}_{t},\omega \right)+\frac{\text{1}}{4\pi }\int_{\text{S},}\sigma_{\text{j}}\left({\vec{\xi }}_{t},\omega \right)\left[\frac{\partial \text{G}}{\partial \text{n}\left({\vec{x}}_{t}\right)}+\text{i}\alpha_{t}{\text{f}}_{t}\text{G}\right]\text{dS}=-\text{i}\omega n_{j}\left({\vec{x}}_{t}\right)$$ (9)

The first order dynamic water pressure is calculated using the linearised Bernoulli equation, given by:


$${\text{p}}^{\left(\text{1}\right)}\left({\vec{x}}_{t},\omega \right)=\text{i}\omega \rho_{t}\phi \left({\vec{x}}_{t},\omega \right)$$ (10)

$${\text{p}}^{\left(\text{1}\right)}\left({\vec{x}}_{t},t\right)={\text{p}}^{\left(\text{1}\right)}\left({\vec{x}}_{t},\omega \right){\text{e}}^{-\text{i}\omega t}=-\rho_{t}\frac{\partial \left[\phi \left({\vec{x}}_{t},\omega \right){\text{e}}^{-\text{i}\omega t}\right]}{\partial \text{t}}=\text{i}\omega \rho_{t}\phi \left({\vec{x}}_{t},\omega \right){\text{e}}^{-\text{i}\omega t}$$ (11)

The *j*th force generated by the *k*th unit amplitude water tank motion-induced wobble with respect to the LTA origin is given by the following equation:


$${\text{F}}_{tjk}=-\text{i}\omega \mu \rho_{t}\int_{{\text{S}}_{t}}\phi_{k}n_{j}\text{dS}$$ (12)

Where *μ* is the compartment permeability, characterised by the proportion of the volume of water in the compartment to the volume of the whole water tank, which takes the value of 1 in the calculation process, but the actual platform compartment contains various beams or tendons. Finally, the force of the water in the internal tank will be obtained and then superimposed on the force of the platform for calculation. The specific and fascinating derivation can be found in the method manual.

### Deep learning framework

For platforms like “Guo Hai Shi 1” irregular tank geometries demand tailored hydrodynamic models to capture tank-hull interactions accurately. Moreover, real-time prediction of roll or pitch—and integration with ballast adjustment strategies—remains underdeveloped. To address these issues, this study: Adopts a numerical simulation framework calibrated to “Guo Hai Shi 1” tank geometries, identifying sensitive roll frequencies and quantifying damping effect across ballast ratios. Employs a CNN-BiLSTM-Attention network to forecast platform roll or pitch time series, enabling early warning when responses may exceed safety thresholds and informing control actions. Subsequent efforts will focus on conducting in-depth research and formulating a ballast adjustment strategy grounded in predictive output control theory, aiming to actively mitigate undesirable platform movements.

The CNN_BiLSTM_Attention model is a hybrid deep learning framework that integrates:

Convolutional Neural Networks (CNN) to extract local features from time-series data [15]. The Convolutional Neural Network (CNN) is a deep learning architecture specifically designed for processing grid-structured data. By leveraging local connectivity and weight-sharing mechanisms, CNNs significantly reduce computational overhead while enhancing spatial hierarchical feature extraction capabilities [16]. These networks automatically learn multi-level features from raw data, compress information via pooling and convolutional operations, suppress irrelevant noise, and improve model robustness [17].

Bidirectional Long Short-Term Memory (BiLSTM) networks to capture long-term dependencies in both forward and backward directions [18].The Bidirectional Long Short-Term Memory network (BiLSTM) is an enhanced recurrent neural network (RNN) architecture designed to address the gradient vanishing problem inherent in traditional RNNs during long-sequence modeling [19]. Its core feature lies in a bidirectional information propagation mechanism that simultaneously captures forward and backward temporal dynamics, significantly enhancing the model’s ability to model temporal dependencies [20]. BiLSTM has demonstrated exceptional performance in natural language processing and speech recognition, and recent applications in time-series prediction, anomaly detection, and fault diagnosis highlight its unique advantages. Future research will explore its deployment in platform anchor-system monitoring.

Attention Mechanism to focus on key information, improving model interpretability and prediction accuracy [21]. In floating platform motion prediction, the contributions of features at different time nodes to forecasting targets vary significantly (e.g., transient wave impact peaks, resonant phase shifts). Traditional time-series models treat all historical features equally, risking the dilution of critical information by noise. The Attention Mechanism addresses this by dynamically assigning feature weights, enabling the model to focus on temporally relevant segments [22].

This combination makes the model particularly effective for handling time-series data, enabling it to learn complex dependencies and improve motion prediction accuracy.

## Case study

**Fig 2:**
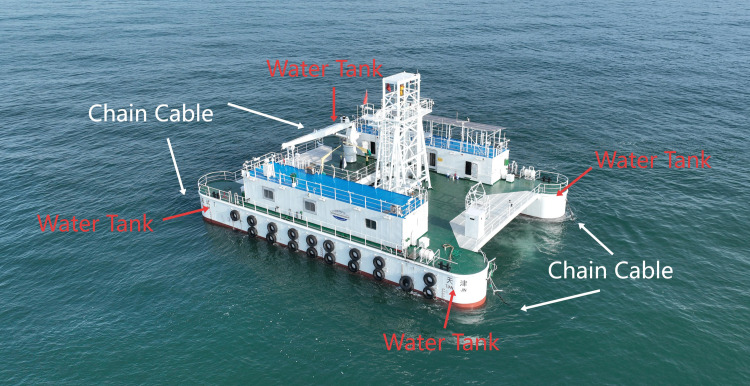
The “Guo Hai Shi 1” offshore test platform.

The “Guo Hai Shi 1” offshore test platform is a steel non-powered floating mooring platform with a gross tonnage of 432 t, adopting a double-floating hull type, with a hull length of 30 m, a width of 21 m, a depth of 4.5 m, a design draught of 2.2 m, and the actual deployment draught of 2.07 m, and the design ballast tanks are located in the semicircular bowsprits and stern-tip tanks around the platform. The actual structure of the platform is shown in Fig 2.

### Sea area information

The National Ocean Technology Center of China conducted year-round wave observations in the waters where the platform was deployed, utilizing wave sensors mounted on multi-parameter integrated buoys. Additionally, the flow velocities of both major and minor tidal currents were measured using Acoustic Doppler Current Profilers [23]. Based on the field observation data and historical records, the wind, wave, and current parameters for different return periods in the area were estimated. The estimation results are presented in Table 1 [24].

**Table 1:** The calculation results of different recurrence periods of wind and waves.

Return period (year)	Significant wave height (m)	Significant wave period (s)	Maximum wind speed (m/s)	Maximum current speed (m/s)
100	6.3	12.72	29.70	1.29
50	6.0	11.98	27.47	1.23
25	5.7	11.24	25.98	1.20
20	5.5	10.99	24.50	1.18
10	5.2	10.25	22.20	1.16
5	4.8	8.39	19.00	1.13
2	4.1	8.27	16.19	1.10

### Mooring system

The mooring system is a critical component of floating offshore platforms, and extensive research has been conducted on mooring line configurations [25,26], anchor fixing methods [27], and mooring chain safety [28]. Offshore platforms are subjected to combined environmental loads from wind, waves, and currents acting from arbitrary directions. To provide restoring forces in all directions, the mooring lines in a mooring positioning system are typically arranged in a radially symmetric configuration in some medium and large platforms.

**Fig 3:**
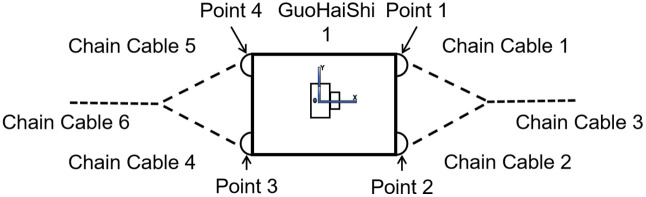
Mooring system.

The “Guo Hai Shi 1” platform employs two 32 mm-diameter Y-shaped AM2 mooring chains, with the upper segments measuring approximately 50 m each and the lower segments extending 150 m as shown in Fig 3. A slack mooring system is adopted, with the key parameters of the mooring chains summarized in Table 2.

**Table 2:** The chain cable parameters.

Parameter	Design size
Number	2
Length (m)	250
Diameter (m)	0.032
Stiffness, EA (kN)	245000
Initial Tension (kN)	13
Expectrd Tension (kN)	583

### Numerical calculation model

#### Platform-mooring coupled hydrodynamic model.

**Fig 4:**
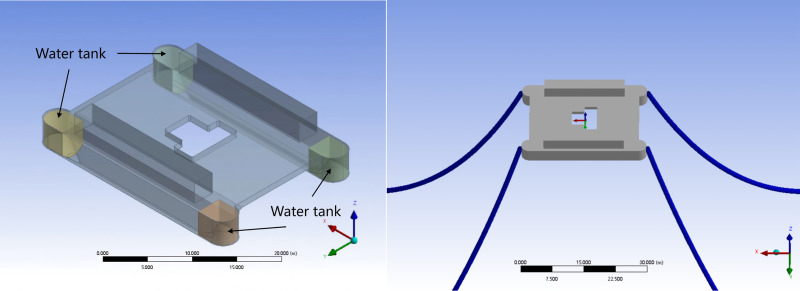
Finite element model of the “Guo Hai Shi 1” offshore test platform.

When constructing the platform’s numerical model, the software does not compute the internal structure or material parameters of the “Guo Hai Shi 1” platform. Therefore, only the wetted surface model needs to be established, with the designed draft depth of 2.1 m as the reference waterline. As shown in Fig 4, the “Guo Hai Shi 1” offshore test platform features a twin-hull structure, with a total of four ballast water tanks located at the bow and stern ends.

Since the actual Y-shaped mooring chain arrangement of the platform cannot be directly simulated so that an equivalent modeling approach is adopted. In the simulation, four 165 m-long mooring chains are used to connect the anchor points to the platform attachment points, approximating the behavior of the real mooring system. The primary dimensional parameters and hydrostatic properties of the platform are listed in Table 3.

**Table 3:** Main dimensional parameters and hydrostatic parameters of the platform.

Parameter	Value
Total length (m)	30
Waterline length (m)	30
Moulded breadth (m)	21
Demi-body width (m)	3.6
Demi-body center distance (m)	17.4
Moulded depth (m)	4.5
Draft depth (m)	2.1
Gross tonnage (t)	432
Actual displacement (t)	305

#### Wetted surface model validation.

To enhance the accuracy of the model’s fundamental parameters, a simulation in the time domain is conducted. The platform model is first tilted to an initial angle of 7.5° and then released to undergo free decay. No external disturbances are introduced, allowing the system to evolve solely under the influence of gravity until a steady-state condition is achieved. Once the platform stabilizes, the water levels at four reference points on the port and starboard sides are recorded and compared with the actual draft of the platform. The locations of these waterline reference points are shown in Fig 3, and the corresponding comparison results are presented in Table 4.

**Table 4:** Comparison of model draft and actual draft.

Point	Physical (m)	Model (m)	Add Anchor Chain (m)	Difference 1 (m)	Difference 2 (m)	Difference Percent
Point 1	2.020	2.037	2.066	0.017	0.046	0.854%
Point 2	1.950	1.972	2.002	0.022	0.052	1.142%
Point 3	2.130	2.157	2.184	0.027	0.054	1.265%
Point 4	2.200	2.222	2.248	0.022	0.048	0.997%

As shown in Table 4, the calculated draft heights at the four reference points are highly consistent with the actual draft of the platform, with a maximum deviation of less than 1.3%. Additionally, while the model’s draft is set to 2.1 m, observations indicate that the actual platform’s draft is slightly less than 2.1 m. Additionally, the classification certificate issued by the China Classification Society for the “Guo Hai Shi 1” platform was reviewed. According to the certificate, the natural pitch period of the platform is 3.11 s, and the metacentric height is 50.129 m. When the central lifting frame is fully lowered (100% down), the center of gravity is located at 3.55 m, and when fully raised (100% up), the center of gravity shifts to 4.275 m. In comparison, the corresponding parameters obtained from the wet model test indicate a natural roll period of 3.21 s, a metacentric height of 50.06 m, and a center of gravity position of 3.79 m when the lifting frame is in a neutral position (neither raised nor lowered). Minor discrepancies are observed between the model and the actual platform in terms of these parameter, all of which remain within acceptable limits. Therefore, it can be concluded that the hydrostatic parameters of the wetted surface model closely match those of the physical platform, validating its suitability for subsequent hydrodynamic simulations.

#### Design working condition combination.

To study the interaction between ballast water volume variation in ballast tanks and wave loads, as well as to determine the sensitive frequencies of ballast water with specific drainage ratios under wave action, the platform’s oscillatory motion is analyzed under regular waves. As illustrated in Fig 4, rolling motion is defined as rotation about the X-axis, while pitching motion is defined as rotation about the Y-axis. A 180° rotation along the negative X-axis direction is defined as the platform directly facing the incident waves. Four wave periods are selected to represent different sea states, ranging from an extreme once-in-a-century event to a typical third-level daily sea condition, with the corresponding test conditions listed in the following Table 5.

**Table 5:** Regular wave condition combination.

Working Case	Significant Wave Period (s)	Significant Wave Height (m)	Direction (°)
Case1	4.8	1	180°
Case2	6.84	2.5	180°
Case3	8.39	4.8	180°
Case4	12.72	6.3	180°

Additionally, to evaluate the roll-reduction effect of ballast tanks under real sea conditions, irregular waves and wind-current coupling effects should be incorporated to simulate actual scenarios. For the safety of experiment personnel and equipment, the “Guo Hai Shi 1” platform does not conduct offshore experiments beyond sea state level 4. Therefore, three sets of sea state conditions (levels 4, 6, and 8 shown in Table 6) are established to simulate real conditions. The irregular wave spectrum is modeled using a Pierson-Moskowitz (P-M) short-peaked spectrum under a relatively developed sea state.

**Table 6:** Combination of working conditions for irregular waves.

Working Case	Significant Wave Period (s)	Significant Wave Height (m)	Direction (°)	Maximum wind speed (m/s)	Maximum current velocity (m/s)	Sea State Level
Case5	4.8	1	180°	10.00	1	4
Case6	8.39	4.8	180°	19.00	1.13	6
Case7	15.4	12	180°	30	1.29	8

**Fig 5:**
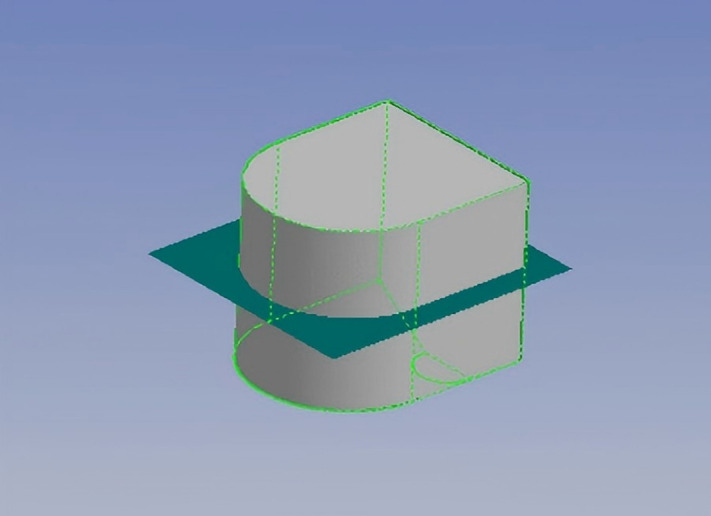
Position of the waterline inside the tanks with 30% displacement added.

Due to the irregular shape of the ballast tanks, using a quantified waterline approach may not be applicable. Thus, based on the actual displacement of the platform, a series of ballast water discharge ratios is considered. Specifically, ballast water is added in increments of 0%, 5%, 10%, 15%, 20%, 25%, and 30% of the total 310 t displacement, evenly distributed across all ballast tanks, to analyze the hydrostatic performance. The detailed values for four selected conditions are presented in the following Table 7, and one of the condition cases is shown in Fig 5.

**Table 7:** Ballast tank water volume setting.

Working Condition	Percentage of Displacement	Total Ballast Water (t)	Single Cabin (t)
Con 1	0%	0	0
Con 2	10%	31	7.75
Con 3	20%	62	15.5
Con 4	30%	93	23.25

#### Hydrostatic parameter analysis of the model.

**Fig 6:**
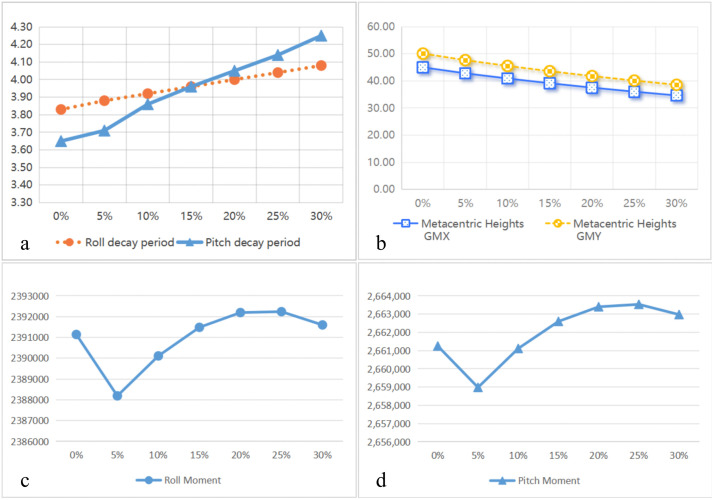
The upper left Fig a is the decay periods in the free decay simulation, the upper right Fig b is the metacentric height, the lower Fig c and d are the restoring moment in the roll and pitch degrees of freedom.

The hydrostatic free decay analysis was conducted for the several different ballast tank models, yielding the variation trends of the platform model’s restoring moment, metacentric height, and natural period, as shown in the Fig 6. The results indicate that as the ballast water volume increases, both the roll and pitch periods exhibit a slight increasing trend. However, the increase is minimal, with a maximum difference of only 0.2 s for roll and 0.6 s for pitch. This suggests that adding a certain amount of ballast water does not significantly affect the platform’s free decay oscillation period. On the other hand, the metacentric height along both the X and Y-axes decreases as the ballast water volume increases, while the restoring moment follows a trend of initially decreasing, then increasing, and decreasing again at a 20% ballast water ratio. From a stability perspective, adding more ballast water does not necessarily improve stability indefinitely.

## Results and discussion

### Regular wave response

**Fig 7:**
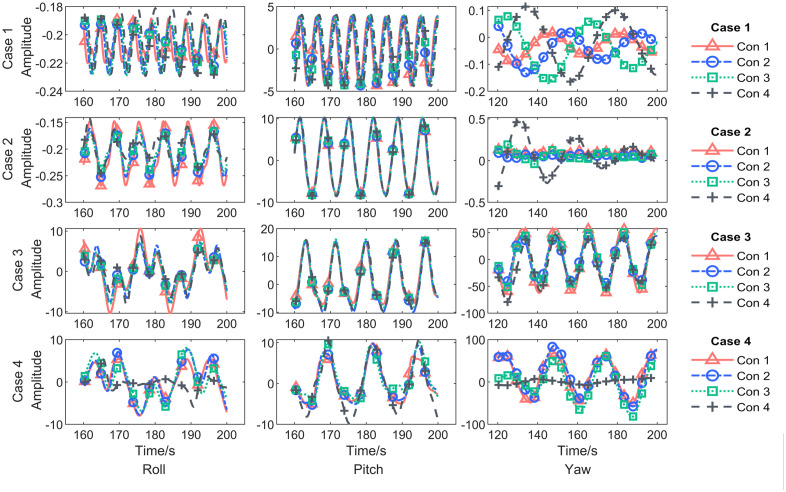
The time history curve of the platform’s response motion under regular wave excitation.

According to the hydrostatic parameter analysis results, these four conditions in Table 7 were selected for time domain analysis, both set the simulation step size to 0.01 s, the data output 0.01s, and the simulation duration 200 s. Some of the results are shown below Fig 7.

**Fig 8:**
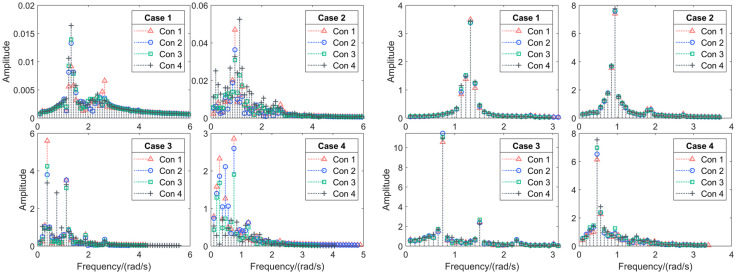
The component spectrum in the roll and pitch degrees of freedom.

**Fig 9:**
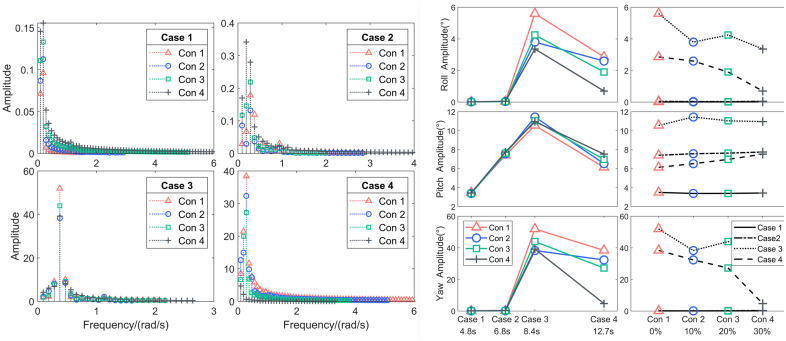
The component spectrum in the yaw degrees of freedom and the maximum statistics.

The component spectrum was obtained through component separation of the simulation results, as shown in the Figs 8 and 9, followed by an extreme value statistical analysis.

When the high-frequency, low-amplitude regular wave of Case 1 was set as the incident load, the platform—when directly facing the waves—exhibited a significant response only in the pitch direction, while the roll and yaw degrees of freedom showed negligible wave-induced motion. In terms of trends, the response component amplitude for the four different ballast water conditions initially decreases and then increases with wave frequency, but the numerical differences are small. The maximum pitch response amplitude occurred in Con4, reaching approximately 3.43°.

In Case 2, the platform’s motion response across the four ballast water conditions followed a similar pattern to Condition 1 but with amplitudes approximately twice as large and a maximum value of 7.76°.

When Case 3 was set with a mid-frequency, mid-amplitude regular wave as the external excitation, the platform exhibited significant responses in all three oscillation degrees of freedom. Roll Motion: The largest response component spectrum amplitude appeared in Con1, with a peak value of 5.60°, while the smallest occurred in Con4 at 3.36°. Pitch Motion: The highest amplitude was observed in Con2 at 11.45°, while the smallest was in Con1 at 10.56°. Additionally, another distinct peak appeared at the platform’s pitch frequency, indicating a more pronounced resonance effect compared to other conditions. Yaw Motion: The maximum response occurred in Con1, reaching 51.94°, while the minimum was in Con4 at 39.22°. These results suggest that when the wave period is approximately twice the platform’s natural period, the platform exhibits the most sensitive response with significantly larger motion amplitudes.

When the incident load was the low-frequency, high-amplitude waves of Case 4, none of the four ballast water configurations exhibited resonance with the waves. Among the response components spectrum: Roll Motion: The maximum response amplitude occurred in Con1, while the minimum was in Con4. Pitch Motion: The largest response amplitude was observed in Con4 at 7.53°, while the smallest was in Con1 at 6.14°. Yaw Motion: The most significant response appeared in Con1, while the smallest was in Con4.

These results indicate that under high sea states, although Con4 exhibited a relatively large pitch response amplitude in the incident wave direction, the ballast tanks effectively suppressed motion in the roll and yaw degrees of freedom.

By analyzing the maximum response components with wave conditions(wave period) as the horizontal axis, it is observed that as the wave period increases, significant response values in the roll and yaw degrees of freedom only occur under higher sea states. The pitch frequency response amplitude, however, follows a trend of first increasing and then decreasing, with the most sensitive frequency appearing at twice the platform’s natural period.

When analyzing the maximum response components with ballast tank liquid volume as the horizontal axis, it is found that as the liquid volume increases, the frequency response amplitudes of roll and yaw under high sea states show a decreasing trend. In contrast, the pitch response amplitude exhibits a gradual increase, although the variation remains relatively small.

In summary, the ballast water tanks have a relatively limited roll-reduction effect on the platform’s primary response degree of freedom under wave excitation, i.e., the direction of the frontal facing wave. However, they effectively suppress coupled responses in other degrees of freedom, allowing the platform to maintain a dynamic equilibrium where motion is primarily confined to a single degree of freedom.

### Irregular wave response

**Fig 10:**
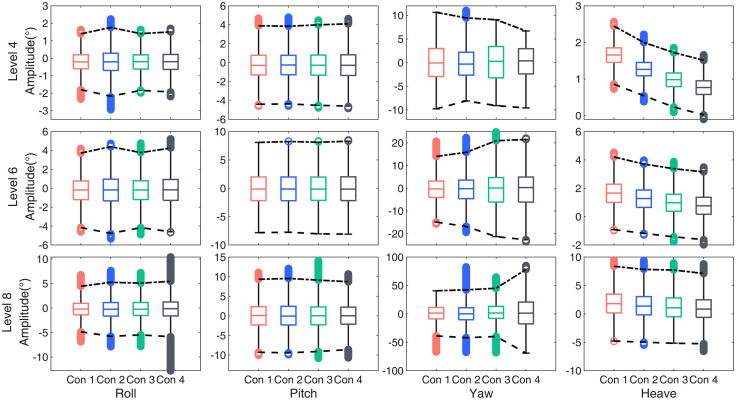
The box plot of the platform’s response motion under irregular excitation.

Using the Pierson-Moskowitz (P-M) short-peaked irregular wave spectrum as the incident wave load, a box plot analysis was conducted on the simulation data, as shown in the Fig 10. The whiskers of the box plot represent the expected variation of the data and are used to determine the maximum and minimum values for statistical analysis while avoiding extreme outliers.

For the primary response degree of freedom—pitch motion—the floating platform’s response under four different ballast water conditions was analyzed across three sea states:

In sea state level 4, the pitch response amplitude initially decreases and then increases. Compared to the no-ballast condition (Con1), Con2 reduces the pitch response by 1.26%, whereas Con3 and Con4 increase it by 2.5% and 5.3%, respectively.

In sea state level 6, all conditions show an increasing trend, with Con2, Con3, and Con4 increasing by 2.02%, 0.46%, and 2.71%, respectively.

In sea state level 8, the response first increases and then decreases, where Con2 increases by 2.31%, while Con3 and Con4 decrease by 2.17% and 6.54%, respectively.

Due to the short-peaked irregular wave spectrum, which introduces wave excitation not only in the primary direction but also in other directions, the response in coupled degrees of freedom does not follow the same trend observed under regular waves.

Roll Motion: Across all three sea states, the roll response amplitude exhibits a general increasing trend: Con2 shows the largest increase (20.85%) in sea state level 4. Con3 has a maximum increase of 13.51% in sea state level 8. Con4 reaches its peak increase (20.82%) in sea state level 8. These results indicate that under short-peaked irregular wave conditions, platforms with different ballast water configurations may fail to suppress the response amplitude in coupled degrees of freedom.

Yaw Motion: In sea state level 4, the response amplitude follows a decreasing trend. In sea states 6 and 8, the response amplitude increases.

A statistical analysis of heave motion was performed, as shown in the figure below. The results demonstrate that adding ballast water effectively reduces the platform’s vertical displacement amplitude, though this ability weakens as sea conditions deteriorate. In sea state level 4, Con2 provides the best wave resistance, reducing heave motion by a maximum of 8.34%. In sea states 6 and 8, Con4 performs best, reducing heave motion by 6.85% and 6.06%, respectively.

These suggest that while ballast water does not significantly mitigate responses in coupled degrees of freedom under short-peaked irregular waves, it plays a notable role in reducing heave motion, helping to stabilize the platform under worsening sea conditions.

### Prediction of time series

Based on the simulation data obtained from the previous analysis, a CNN_BiLSTM_Attention model was constructed to perform time series prediction and regression analysis for the pitch motion of the “Guo Hai Shi 1” platform. This model could be used for real-time monitoring of platform motion after deployment, predicting whether the pitch or roll angle exceeds safety thresholds, and supporting ballast water adjustment strategies based on control theory to mitigate undesirable motions.

The dataset generated from hydrodynamic analysis was processed using a sliding window technique, ensuring that sequential dependencies were maintained. Both features and targets were individually normalized to improve model convergence and stability. The features independent variable are “Ballast Water”, “Current Force”, “Wind Force”, “Diffraction Force”, “Linear Damping Force”, “Drift Force”, “Mooring Force”, “Radiation Force”, “Sea State” and the target dependent variable is pitch motion. The model accommodates motions with varying degrees of freedom; hence, pitch movement is selected for illustrative purposes.

**Fig 11:**
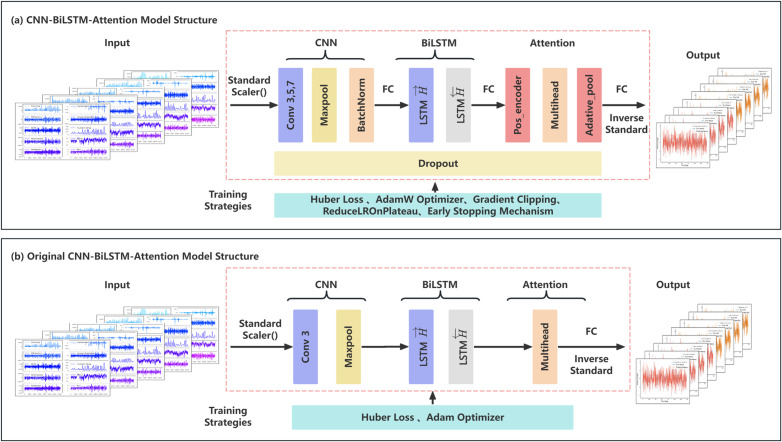
The algorithm architecture of CNN_BiLSTM_Attention model.

To enhance the performance of the original CNN_BiLSTM_Attention model which refer to algorithm architecture initially constructed based on this paper [20], several optimizations were implemented, as summarized in the following, and the algorithm architecture is shown in Fig 11.

1. Multi-Scale CNN for Feature Extraction

Utilized three parallel convolutional kernels (sizes 3, 5, and 7) to capture multi-scale temporal dependencies.

2. Enhanced Bidirectional LSTM (BiLSTM)

Dropout regularization was introduced to prevent overfitting and improve generalization.

3. Multi-Head Attention Mechanism

Replaced the original single-head attention with multi-head attention to enhance feature interaction capabilities and better capture long-range dependencies.

4. Deep Fully Connected Layers

Added residual connections to improve gradient flow and prevent vanishing gradients. Incorporated additional nonlinear layers to increase feature learning capacity.

To improve model robustness and stability, the following training strategies were deployed in Table 8.

**Table 8:** Robustness optimization strategy.

Technique	Purpose
Huber Loss	More robust to outliers, balancing MSE and MAE behavior.
AdamW Optimizer	Regularization with weight decay, preventing overfitting.
Gradient Clipping	Prevents gradient explosion in deep networks.
Dynamic Learning Rate (ReduceLROnPlateau)	Automatically adjusts learning rate when loss plateaus.
Early Stopping Mechanism	Stops training when validation loss stops improving, preventing overfitting.

To assess the performance of the model, the evaluation metrics used include:MAE (Mean Absolute Error), RMSE (Root Mean Square Error), R² Score (Coefficient of Determination) and Comparison of Predicted vs. Actual Values.

**Fig 12:**
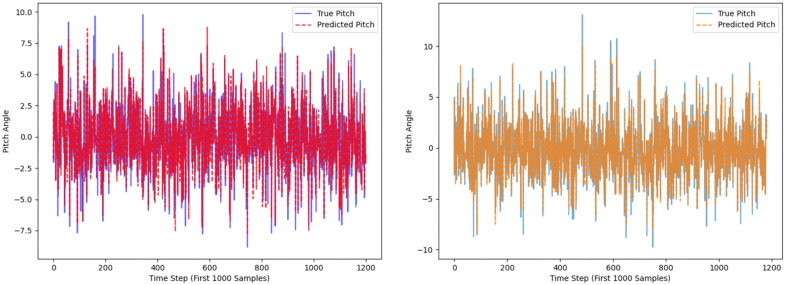
The time-history curve of actual values versus predicted values in the test set.

**Fig 13:**
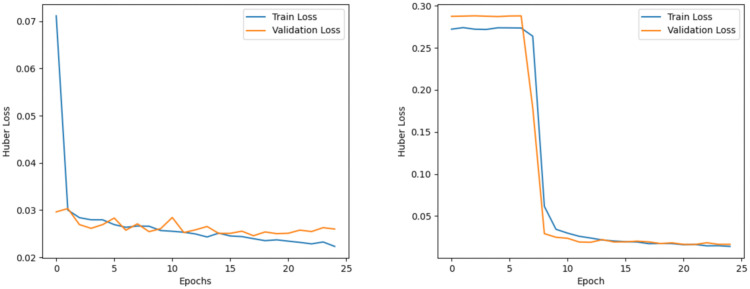
The loss function of the training set and validation set for each training epoch.

**Fig 14:**
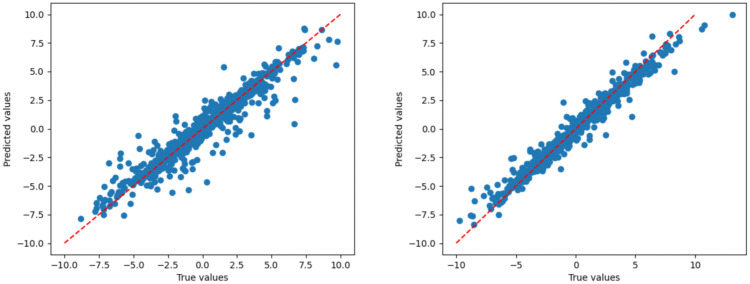
Regression curve of the validation set (True values vs Predicted values).

The comparison results between the original CNN_BiLSTM_Attention model (Left Fig) and the optimized CNN_BiLSTM_Attention model (Right Fig) are shown in the figures, including that Time-history curves of predicted vs. actual values (Fig 12), huber loss curves during training epoch (Fig 13), regression prediction curves (Fig 14).

The rectified linear unit (ReLU) activation function is consistently employed, and 2 layers of LSTM neurons and 128 LSTM hidden units are set. Concurrently, the dataset is partitioned into training, validation, and testing subsets following a 7:2:1 ratio. By setting 25 training epochs with a batch size of 64, the optimized model achieved significant performance improvements compared to the original model. As shown in Table 9, the R² score of the optimized model improved by 4.82%, while the MSE, RMSE, and MAE were reduced by 51.45%, 30.31%, and 24.52%, respectively, which suggests that the optimised model excels in capturing data trends and suppressing overall and extreme errors. However, there is still room for improvement in reducing generally small errors. These results indicate that the optimized model significantly outperforms the original model in both accuracy and generalization ability. The feature selection and target variables in this study serve as a preliminary analysis to validate the feasibility of the model. In future research, a more comprehensive investigation will be conducted, which will be further analyzed to enhance the robustness and practical applicability of the model, including:

**Table 9:** Difference comparison.

Comparative parameters	Original model	Optimized model	Performance improvement
R² Score	0.9214	0.9658	↑ 4.82%
Mean Squared Error (MSE)	0.5879	0.2854	**↓** 51.45%
Root Mean Squared Error (RMSE)	0.7667	0.5343	**↓** 30.31%
Mean Absolute Error (MAE)	0.4224	0.3188	**↓** 24.52%

- Detailed exploration of model optimization techniques
- Wavelet denoising for high-noise load data
  - Integration of real-time sensor feedback, incorporating environmental hydrodynamic features (e.g., wave height, wave period, wind fields)
- Incorporating multi-degree-of-freedom hydrodynamic simulation data
- Comparison with real-scale experimental data from tank tests

## Conclusions

This study investigates the influence of ballast tanks on the stability performance of the “Guo Hai Shi 1” floating platform using the internal tank theory. The platform is equipped with four independent empty tank located at the bow and stern, which serve as anti-roll tanks. By varying the volume/weight of the liquid inside the tanks, the hydrostatic parameters were computed, and the platform’s motion response amplitude under regular and irregular waves was analyzed to evaluate the anti-rolling capability of the ballast tanks. The key conclusion are as follows:

1. Hydrostatic parameters: Adding ballast water had minimal impact on the platform’s hydrostatic parameters. Roll and pitch natural decay periods increased slightly, and the metacentric height decreased. The restoring moment exhibited a non-monotonic variation with ballast volume.
2. Response under regular waves: Under regular-wave excitation, a strong significant resonance effects occurred at a wave period approximately twice the platform’s natural pitch period. The peak value of pitch response spectrum first increased and then decreased with increasing wave period. With more ballast water, the roll and yaw response amplitudes under high sea states were reduced. Overall, the ballast tanks had limited effect on the primary motion degree of freedom but significantly suppressed coupled responses in other degrees of freedom.
3. Response under irregular waves: Under irregular-wave excitation, the ballast tanks reduced pitch response amplitudes only in higher sea states and had negligible wave resistance effect on roll and yaw motions. However, for heave motion, the ballast tanks effectively reduced the platform’s wave-induced vertical displacement amplitude, indicating improved wave-following performance..
4. Neural network prediction: A CNN-BiLSTM-Attention neural network model was developed using the hydrodynamic simulation dataset to predict the platform’s pitch response time series. The optimized model demonstrated good feasibility in capturing the platform’s motion response.

These analysis suggests that the four independent ballast tanks of the “Guo Hai Shi 1” did not significantly enhance platform’s anti-rolling or wave resistance capabilities, but markedly improved wave-following performance. To enhance their effectiveness, a U-shaped anti-roll tank concept could be adopted, where the fore and aft ballast tanks on each side are interconnected to form a unified system. Therefore, further numerical simulation analyses and physical model validation studies of this type of anti-roll tanks will be carried out in the subsequent work, and the CNN_BiLSTM_Attention algorithm will be investigated in more detail with the data from these results. This predictive capability can facilitate early warnings when dynamic responses approach safety thresholds and inform real-time control measures. Building upon these predictions, future studies will also explore the development of a ballast water adjustment strategy based on predictive output control theory, aiming to actively suppress undesirable platform motions.

Nevertheless, certain limitations of the present study should be acknowledged. This analysis is conducted based on a specific platform model and relies exclusively on hydrodynamic simulation data, without corroboration from experimental or field measurements. Despite this, the proposed methodology demonstrates meaningful potential for application. Future studies should consider physical model experiments or alternative other approaches to validate and potentially generalize these findings.

## References

[1] Naziri HAA, Ibrahim AI, editors. Multi-stabilizer devices for marine vessel, design and control–a review. 2022 IEEE 9th International Conference on Underwater System Technology: Theory and Applications (USYS). IEEE; 2022. doi: 10.1109/USYS56283.2022.10072471

[2] Van Slooten M. Mathematical modeling of free-flooding anti-roll tanks [Master thesis]. Delft University; 2014.

[3] İnmeler C. Ballast water management in tankers. Turkey: Dokuz Eylul Universitesi; 2009.

[4] Gawad AFA, Ragab SA, Nayfeh AH, Mook DT. Roll stabilization by anti-roll passive tanks. Ocean Eng. 2001;28(5):457–69. doi: 10.1016/s0029-8018(00)00015-9

[5] Zhang B-L, Han Q-L, Zhang X-M. Recent advances in vibration control of offshore platforms. Nonlinear Dyn. 2017;89(2):755–71. doi: 10.1007/s11071-017-3503-4

[6] Ribeiro e Silva S, Vásquez G, Guedes Soares C, Marón A, editors. The stabilizing effects of U-tanks as passive and controlled anti-rolling devices. International Conference on Offshore Mechanics and Arctic Engineering. American Society of Mechanical Engineers; 2012. doi: 10.1115/OMAE2012-83501

[7] Liu Y, Liu B, Long Y, Liu F. Dynamic analysis of a floating offshore wind turbine with ballast water based on a general framework for external-internal flow coupling. Ocean Eng. 2025;330:121192. doi: 10.1016/j.oceaneng.2025.121192

[8] Zhang K-D, Wang W-H, Piao T-W, Liu Y-H, Leng S-D, Xiu Y-B, et al. Active anti-rolling characteristics of fluid momentum wheel for cylindrical FPSO under wave conditions. Mar Struct. 2025;101:103773. doi: 10.1016/j.marstruc.2024.103773

[9] Lihua L, Jiming W, Jiguang S, Yang L. Design and simulation investigation of variable period passive anti-rolling tank. Chinese J Ship Res. 2021;16(4):147–54. doi: 10.19693/j.issn.1673-3185.02044

[10] Alujević N, Ćatipović I, Malenica Š, Senjanović I, Vladimir N. Ship roll control and power absorption using a U-tube anti-roll tank. Ocean Eng. 2019;172:857–70. doi: 10.1016/j.oceaneng.2018.12.007

[11] Bernal-Colio VR, Gómez-Goñi J, Cercos-Pita JL. CFD computation of the hydrodynamic torque due to free-surface antiroll tanks with 3D dynamics. SOS. 2020;16(8):879–91. doi: 10.1080/17445302.2020.1787929

[12] Xiao Q. Analysis of hydrodynamic characteristics and development of control system for U-shaped anti-roll tank. SST. 2023;45(10):23–6. doi: 10.3404/j.issn.1672-7649.2023.10.005

[13] Wu Q. Research on U-type anti-rolling tank damping structure based on CFD. Dalian University of Technology; 2018.

[14] Wang C. Research on the roll motion of ocean engineering ships with anti-roll tank in waves. Tianjin University; 2019.

[15] Zhao B, Lu H, Chen S, Liu J, Wu D. Convolutional neural networks for time series classification. JSEE. 2017;28(1):162–9. doi: 10.21629/jsee.2017.01.18

[16] Khan SH, Iqbal R. A comprehensive survey on architectural advances in deep CNNs: challenges, applications, and emerging research directions. arXiv:250316546 [Preprint]. 2025. doi: 10.48550/arXiv.2503.16546

[17] Zhang Y, Zhang H, Li C, Pu T. Review on deep learning applications in power system frequency analysis and control. Proc CSEE. 2020;41(50):3392–406. doi: 10.1016/j.ijepes.2021.107744

[18] Abduljabbar RL, Dia H, Tsai P-W. Unidirectional and bidirectional LSTM models for short‐term traffic prediction. J Adv Transp. 2021;2021(1):5589075. doi: 10.48550/arXiv.1801.02143

[19] Mienye ID, Swart TG, Obaido G. Recurrent neural networks: a comprehensive review of architectures, variants, and applications. Information. 2024;15(9):517. doi: 10.3390/info15090517

[20] Wei Z, Shaohua J, Gang B, Yang C, Chengyang P, Haixing X. A method for sound speed profile prediction based on CNN-BiLSTM-attention network. JMSE. 2024;12(3):414. doi: 10.3390/jmse12030414

[21] Tutek M, Snajder J. Toward practical usage of the attention mechanism as a tool for interpretability. IEEE Access. 2022;10:47011–30. doi: 10.1109/access.2022.3169772

[22] Chaudhari S, Mithal V, Polatkan G, Ramanath R. An attentive survey of attention models. ACM Trans Intell Syst Technol. 2021;12(5):1–32. doi: 10.1145/346505534336375

[23] Wang H, Li M, Lu K, Liu Y, Li S. Hydrodynamic characteristics experimental of anchored offshore test platform under wind wave current coupling. Ship Eng. 2024;46(08):138–47. doi: 10.13788/j.cnki.cbgc.2024.08.18

[24] Li S, Lu K, Wang H, Zheng H. Research on survivability test method for new types of floating offshore platforms under extreme sea conditions. China Meas Test. 2024;50(08):21–60. doi: 10.11857/j.issn.1674-5124.2024040107

[25] Ghafari H, Dardel M. Parametric study of catenary mooring system on the dynamic response of the semi-submersible platform. Ocean Eng. 2018;153:319–32. doi: 10.1016/j.oceaneng.2018.01.093

[26] Rinaldi G, Gordelier T, Sansom M, Johanning L. Development of a modular mooring system with clump weights. Ocean Eng. 2021;223:108536. doi: 10.1016/j.oceaneng.2020.108536

[27] Shen Z, Yuan Z, Li H, Zhu C. Study on the characteristics of a new hybrid mooring system for dual-platform joint operations. China Ocean Eng. 2023;37(3):506–18. doi: 10.1007/s13344-023-0042-2

[28] Wang Y, Han D, Yuan ZJ. Experimental study on anchor chain vibration characteristics under anchor safety vision. China Meas. 2019;45(10). doi: 10.11857/j.issn.1674-5124.2018100100

